# Accidental Ingestion of a NEO-fit Device Component by a Neonate

**DOI:** 10.31486/toj.21.0111

**Published:** 2022

**Authors:** Omotola O. Uwaifo, Ryan Jay Abrigo

**Affiliations:** ^1^ Department of Neonatology, Ochsner Baptist Hospital, Ochsner Clinic Foundation, New Orleans, LA; ^2^ The University of Queensland Medical School, Ochsner Clinical School, New Orleans, LA

**Keywords:** *Foreign-body migration*, *intubation*, *intubation–intratracheal*, *neonate*

## Abstract

**Background:** Endotracheal tube securement devices are used to reduce the incidence of unplanned extubation of intubated patients. We describe the ingestion of part of an endotracheal tube securement device by a neonate to bring awareness of the risk of ingestion or aspiration of endotracheal tube securement device components in this population.

**Case Report:** A 13-day-old, former 31-week gestational age female infant was noted on routine radiologic evaluation to have a foreign body in the gastrointestinal tract. The foreign body was thought to be an artifact or an object overlying the radiologic image. However, review of previous imaging showed the object initially in the posterior pharynx with progressive migration into the gastrointestinal tract. The patient did not have any clinical features of gastrointestinal obstruction and had been tolerating enteral feeds. The infant's endotracheal tube securement had been changed from a NEO-fit device (CooperSurgical, Inc.) to a NeoBar device (Neotech Products) on day of life 5. The diagnosis of the foreign body was made 8 days later. The infant was followed with serial imaging per pediatric surgery recommendations. The foreign body was spontaneously passed via the rectum several days later without incident. Pathology identified the foreign body as a piece of the NEO-fit device.

**Conclusion:** Awareness of the possibility of ingestion or aspiration from this endotracheal tube securement device is important for patient safety.

## INTRODUCTION

Accidental ingestions are a common occurrence in the pediatric population; however, they tend to be uncommon in the neonatal population. In the United States, almost 70,000 exposures to foreign bodies/toys or miscellaneous objects were documented in children ^<^5 years of age in 2019 according to the National Poison Data System annual report.^1^ Various accidentally ingested objects have been described in the pediatric literature, including button batteries, magnets, coins, nails, pins, tacks, toothpicks, and toys.^2^ Accidental ingestions may be asymptomatic or may present with clinical features such as dysphagia, drooling, choking, chest pain, abdominal pain, vomiting, or hematemesis.^3,4^ Accidental ingestions may lead to severe complications, including perforation and extraluminal migration; abscess formation; peritonitis; fistula formation; appendicitis; liver, bladder, heart, and lung penetration; and death.^2^

Endotracheal tube securement devices are used to stabilize endotracheal tubes in intubated patients. We present a case of accidental ingestion of a piece of an endotracheal tube securement device that to our knowledge has not been previously described in the literature. This case is unique as endotracheal tube securement devices are frequently used to prevent adverse events in intubated infants by reducing unplanned extubation. However, in this case, the device was the cause of the adverse event.

## CASE REPORT

The patient was the second born female twin (Twin B) of a diamniotic dichorionic gestation to a 23-year-old, gravida 1 mother. Pregnancy was significant for chronic hypertension and fetal growth restriction. Delivery was at 31^1/7^ weeks of gestation via cesarean section secondary to poor biophysical profile and absent end-diastolic flow. Maternal prenatal laboratory workup was negative for syphilis, hepatitis B, and human immunodeficiency virus.

The birth weight of Twin B was 1,040 kg (ninth percentile). Following delivery, she developed respiratory distress syndrome and was placed on noninvasive nasal positive pressure ventilatory support. By day of life (DOL) 1, she developed progressive hypoxic respiratory failure, necessitating intubation and mechanical ventilation. She required transfer to a higher level of care on DOL 5 because of worsening respiratory failure.

**Figure 1.**
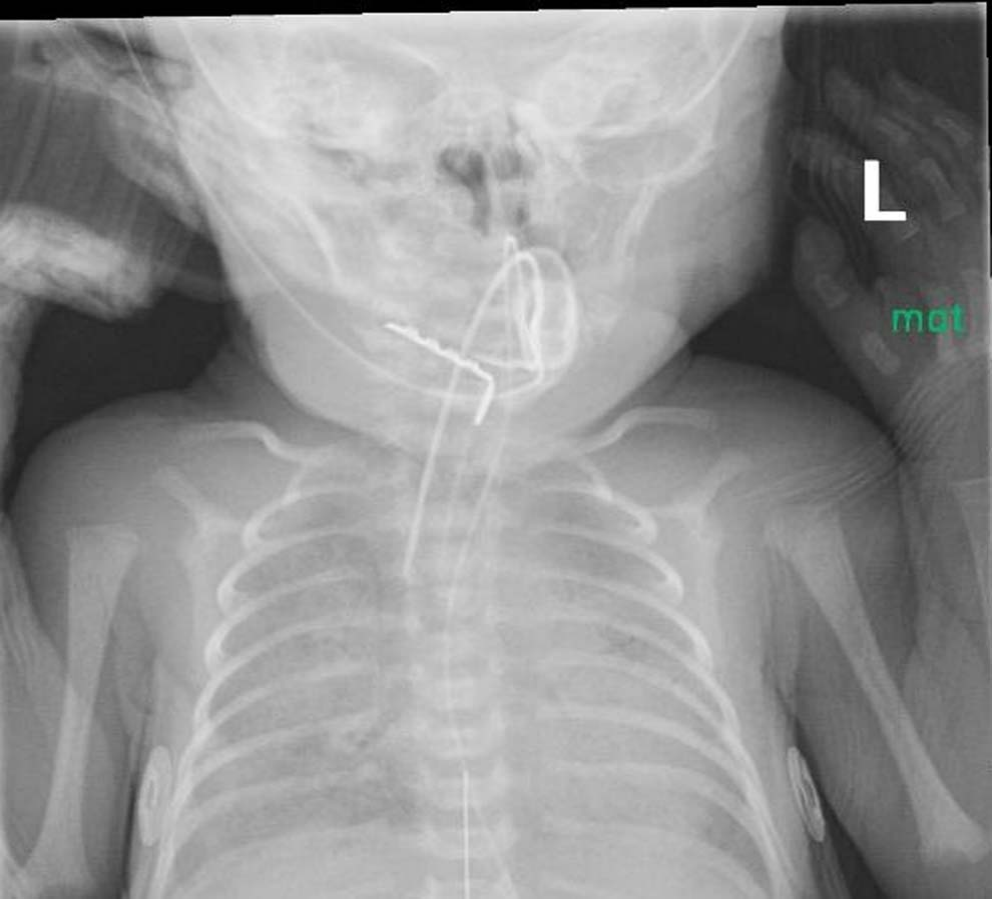
X-ray shows the presence of an L-shaped object in the oral cavity on the day of admission.

**Figure 2.**
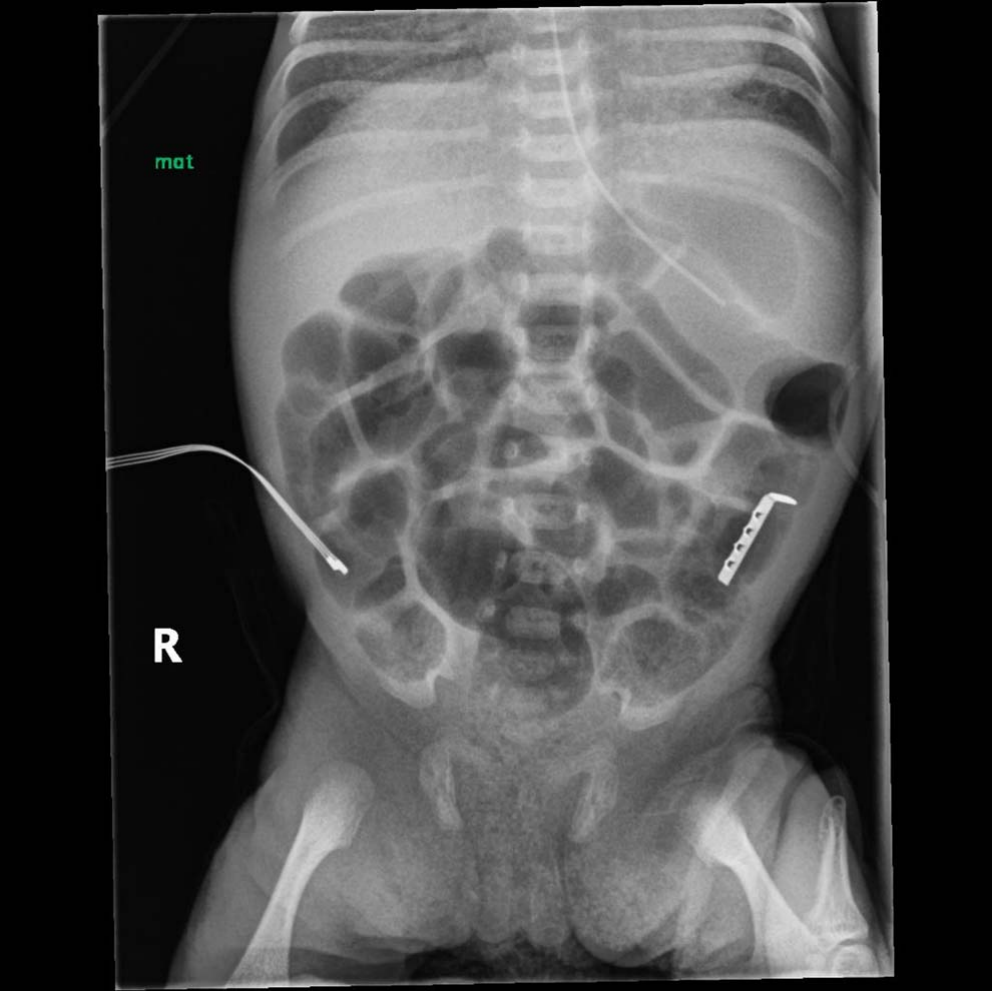
Abdominal x-ray shows the presence of an L-shaped object overlying the left abdomen 11 days after admission.

At the time of transfer to our institution, the infant was orally intubated with a 3.0 endotracheal tube secured with the NEO-fit device (CooperSurgical, Inc.) from the referring hospital. On admission, the endotracheal tube securement device was changed to a NeoBar device (Neotech Products) per our clinical practice. On DOL 13, a routine x-ray revealed a radiopaque L-shaped object projecting over the left abdominal area. Review of prior imaging showed the presence of the object on the day of admission in the oral cavity (Figure 1) with migration on subsequent films into the gastrointestinal tract (Figure 2). Although the object was visualized on prior films, it was thought to be an external artifact and was not mentioned in the imaging reports.

**Figure 3.**
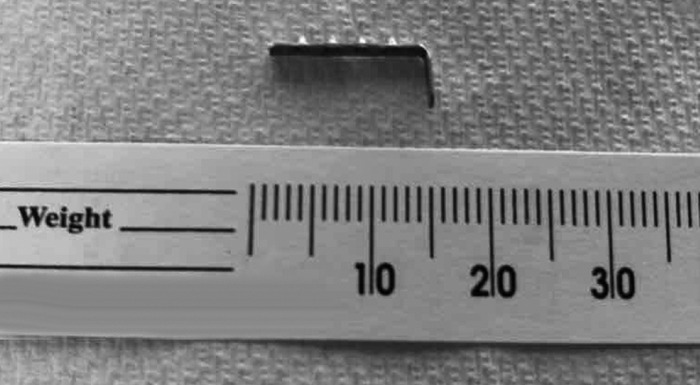
Pathology specimen showing the retrieved metal piece from the NEO-fit device.

At the time of diagnosis, Twin B was hemodynamically stable on mechanical ventilation support. She had no clinical features of gastrointestinal obstruction or trauma such as feeding intolerance, residuals, emesis, or abdominal distention. She was tolerating advancing enteral feeds via gavage of unfortified maternal breast milk with supplemental parenteral nutritional support. She had been passing spontaneous, nonbloody, seedy yellow stools since admission. Examination showed a soft, round, nontender abdomen without organomegaly and with bowel sounds present in all quadrants. Pediatric surgery consultation recommended nonintervention with close clinical monitoring and serial radiologic imaging with the expectation that the foreign object would pass spontaneously via the rectum. Feedings continued to be advanced until full enteral feeds were achieved on DOL 18. Stools were monitored closely to detect the passage of the foreign body. The object was observed to traverse the gastrointestinal tract on serial imaging and was spontaneously passed via the rectum on DOL 17, 12 days after admission, without any complications. The object was sent to pathology and identified as a metallic piece of the hook and loop strap of the NEO-fit endotracheal tube securement device (Figure 3).

## DISCUSSION

Unplanned extubation is defined as any dislodgement of an endotracheal tube from the trachea that is not intentional or the premature removal of the endotracheal tube by the patient or by medical staff during nursing or medical care.^5,6^ Unplanned extubation has been associated with neonatal morbidity and mortality and has been targeted as a patient safety metric.^5^ The incidence of unplanned extubation in the neonatal intensive care unit varies, but a systematic review found a range of incidences from 1% to 80.8%.^6^ One of the risk factors associated with unplanned extubation is poor fixation of the endotracheal tube, which may be caused by loose or wet tape failing to adequately secure the device. The frequency of unplanned extubations associated with poor fixation ranges from 8.5% to 31.0% of total unplanned extubation events.^6^ As a result, a focus of many quality improvement studies has been to find the best and safest practices for endotracheal tube fixation. Studies have shown that changes to endotracheal tube securement can decrease unplanned extubation rates.^7,8^

Various methods have been described to secure endotracheal tubes.^6^ In addition, devices for endotracheal tube securement are commercially available such as the NEO-fit and the NeoBar. A Cochrane review of endotracheal tube securement methods in newborns found a lack of evidence to support the use of any one technique over others.^9^ Because of the diverse assortment of techniques that can be used for endotracheal tube stabilization and the lack of evidence, choosing which method to use may be difficult for clinicians. In many cases, the decision is based on familiarity, availability, or institutional guidelines.

The endotracheal tube securement device used prior to the neonate's transfer was the NEO-fit device that has foam pads affixed to the infant's face and is connected to a plastic base with an adjustable hook and loop strap attached. The adjustable hook and loop strap has metal clips secured to a Velcro strap that mechanically grip the soft material of the endotracheal tube without the use of tape. Each device has 3 metal clips. The metal clips each have 4 raised circular bumps with serrated edges. As shown in our patient, these metal clips can become dislodged and accidentally ingested. The serrated edges of the metal clips could potentially result in mucosal damage if ingested or aspirated. The presence of metal within an endotracheal tube securement device may also contraindicate the use of this device in intubated patients undergoing magnetic resonance imaging (MRI).

We hypothesize that a metal clip from the NEO-fit device became dislodged during the change of endotracheal tube securement on admission. The metal clips are secured to the Velcro strap by tabs on either side and do not fully encircle the strap. The recovered object showed that the tab on one side had broken, resulting in the L-shaped configuration of the piece, and the broken tab likely facilitated the object's dislodgement into the oral cavity where it was ingested. As staff was unfamiliar with the NEO-fit device because it is not used at our institution, the metallic object was not recognized clinically or radiologically as a foreign body on initial evaluations. However, the metallic piece traversed the gastrointestinal system and was expelled without complications.

An alternative to the NEO-fit device is the NeoBar device. The NeoBar consists of a plastic polypropylene bar secured to the infant's face by hydrocolloid tabs. The endotracheal tube is affixed to the NeoBar using tape. The NeoBar consists of one molded piece without parts that can be dislodged. It does not feature any metallic parts and as such can be used on infants undergoing MRI studies.

Because this complication was directly related to a medical device, a Form 3500 was filed with MedWatch, the US Food and Drug Administration Safety Information and Adverse Event Reporting Program, access number MW5099597.^10^

## CONCLUSION

We have described the ingestion by a neonate of a piece of an endotracheal tube securement device that did not result in significant complications. Although our patient had no complications, this case report highlights the potential risk. Future designs of endotracheal tube securement devices should evaluate and mitigate the risk for accidental aspiration or ingestion.

## Notes

*This article meets the Accreditation Council for Graduate Medical Education and the American Board of Medical Specialties Maintenance of Certification competencies for Patient Care and Medical Knowledge.*
